# Choline supported poly(ionic liquid) graft copolymers as novel delivery systems of anionic pharmaceuticals for anti-inflammatory and anti-coagulant therapy

**DOI:** 10.1038/s41598-019-50896-5

**Published:** 2019-10-08

**Authors:** Rafał Bielas, Anna Mielańczyk, Magdalena Skonieczna, Łukasz Mielańczyk, Dorota Neugebauer

**Affiliations:** 1 Department of Physical Chemistry and Technology of Polymers, Faculty of Chemistry, Silesian University of Technology, Strzody 9, 44-100 Gliwice, Poland; 2 Biosystems Group, Institute of Automatic Control, Faculty of Automatics, Electronics, and Informatics, Silesian University of Technology, Akademicka 16, Gliwice, Poland; 3 Biotechnology Centre, Silesian University of Technology, Krzywoustego 8, Gliwice, Poland; 4 Department of Histology and Cell Pathology, School of Medicine with the Division of Dentistry in Zabrze, Medical University of Silesia, Jordana 19, 41-808 Zabrze, Poland

**Keywords:** Bioconjugate chemistry, Drug delivery

## Abstract

New type of carriers based on grafted poly(ionic liquid)s was designed for delivery of ionically attached salicylates (Sal). Choline derived ionic liquid monomeric units were successfully introduced with various content in the side chains by the controlled radical polymerization. Properly high amounts of ionic pharmaceutics in the polymer systems were achieved by the well-fitted length and grafting degree of the side chains. In aqueous solution the graft copolymers were self-assembled into the spherical superstructures with sizes up to 73 nm. Delivery studies showed “burst” release within 4 h, after that it was slower yielding ~70% of released drug within 80 h. Proposed nanocarriers supported low toxicity against human cells (NHDF and BEAS-2B), anti-inflammation activity evaluated with the use of pro-inflammatory interleukins (IL-6 and IL-8) and antibacterial activities towards *E. coli*. Adjustment of ionic drug content by structural parameters of graft copolymers, including grafting degree and graft length, are advantageous to tailor nanocarriers with self-assembly properties in aqueous media. Effective release process by ionic exchange and biological activity with low toxicity are promising for further development of this type of drug delivery (DDS).

## Introduction

Grafted copolymers, including molecular brushes, are materials with unique properties because of extraordinary topology, what makes them one of the most desirable products of macromolecular engineering^1^. There are three main approaches to obtain such polymers, namely *grafting-from*, *grafting-to* and *grafting-through*, which can provide cylindrical polymer brushes by building side chains on the multifunctional macroinitiator (usually modified poly(2-hydroxyethyl methacrylate)^2^), attaching monofunctionalized chains to the multitelechelic polymer or (co)polymerization of macromonomer(s), respectively^3^. These materials have found a broad spectrum of applications from self-repairing surfaces^4^ to sensors^5^ and drug carriers^6^. Cylindrical brushes with polyether side chains^7^ are one of the most investigated systems for delivery of biologically active substances like indomethacin^8,9^ or doxorubicin^10,11^. Other examples have been reported for grafted copolymers with biodegradable polyester side chains^12,13^, or pH-sensitive weak polyelectrolyte side chains like poly((meth)acrylic acid)^14–16^, polyvinylpyridine^17^ and polyethyleneimine^18,19^. The brushes based on biopolymer backbone, like chitosan^20^, or protein^21^ were also applied as drug carriers^22,23^. Such materials ensure exceptional colloidal stability, low cytotoxicity and can be effective gene transfection agents.

Group of nonlinear polymers that gains special interest nowadays are grafted poly(ionic liquid)s^24^, whereas their linear analogs have already found a variety of applications^25^. The poly(ionic liquid)s are usually grafted on surfaces, like modified silica^26^ to design materials for chromatography^27^ and membranes for nanofiltration^28^ or glass for anti-icing coatings^29^. Among a few examples of cylindrical brushes, poly(1-butyl-3-vinyl imidazolium bromide) grafted on poly(vinyl chloride) as an additive improving hydrophilic and antifouling properties of membranes^30^, poly(3-acrylamidopropyl trimethylammonium chloride) on pullulan for waste water treatment processes and drug delivery^31^, have been described.

Expanding the applicability of graft copolymers, including poly(ionic liquid) cylindrical brushes, here we report the synthesis of salicylate anion (Sal) bearing poly(2-(trimethylammonium)ethyl methacrylate) grafted from polymethacrylates as novel drug delivery systems (DDS). Previously, we have investigated linear statistical copolymers of this ionic monomer (also known as choline methacrylate (ChMA)), which was modified with Sal both before or after polymer synthesis^32,33^. Our interest in salicylates was focused due to their antibacterial and anti-inflammatory activities, which are already used in wound healing, diabetes, arthritis, and cancer therapies. However, these medical applications require the delivery of therapeutic doses in stable level^34,35^, which can be maintained by the use of polymeric carriers, as it was observed for salicylate admixtured into poly(anhydride-ester) matrices^36^. In our expectation the application of grafting strategy for copolymers of ionic liquid choline methacrylate should improve drug-carrier ratio and may influence on the release profiles. In this case the Sal contained ionic liquid methacrylate monomer (ChMA/Sal) was copolymerized with methyl methacrylate using multifunctional macroinitiator by controlled radical polymerization ATRP. The resulted in three series of new ionic graft copolymers were varied by composition of side chains (ratios of ionic to nonionic units) and degree of grafting (depending on the amount of initiating moieties in backbone). The self-assembling nanoparticles of amphiphilic polymers were tested by light scattering and electron microscopic techniques. The selected graft copolymers were studied to estimate cytotoxicity towards human cells by MTT assay, as well as the antibacterial and anti-inflammatory activities were evaluated.

## Materials and Methods

### Materials

Methyl methacrylate (MMA) and 2-(hydroxyethyl) methacrylate (HEMA) (both Alfa Aesar) were desiccated using 4 Å molecular sieves (Chempur). [2-(methacryloyloxy)ethyl]trimethylammonium chloride (ChMA/Cl, 80% aq. solution, Sigma-Aldrich) was concentrated to a constant weight by water evaporation. Copper(I) bromide, copper(I) chloride, 2,2′-bipyridine, sodium salicylate (NaSal), tetrahydrofuran (THF), *N,N,N*′*,N*″*,N*″-pentamethyldiethyltriamine (PMDETA) and ethyl 2-bromoisobutyrate (EBiB) were obtained from Sigma Aldrich. Methanol, acetone, DMF, were bought from Chempur.

#### Synthesis of macroinitiators (example for polymer I-Ia)

The mixture of comonomers HEMA (1,4 mL, 11.5 mmol) and MMA (3.8 mL, 35.5 mmol), anisol (0.6 mL), dNbpy (47.27 mg, 0.11 mmol) and CuBr catalyst (7,98 mg, 0.056 mmol) was freeze–pump–thawed twice and the EBiB initiator (11 µl, 0.075 mmol) was added to start reaction at 70 °C. The exposure to air has stopped the reaction after 4 hours. The polymer solution was purified from copper catalyst by passing through a column filled with neutral Al_2_O_3_, then precipitated in diethyl ether and vacuum dried. ^1^H NMR in Fig. S1 (DMSO-d_6_, δ, ppm): 4.87 (1H, –CH_2_–O**H**), 3.91 (2H, –C**H**_**2**_–OH), 3.68–3.35 (2H, –COO–C**H**_2_– and 3H, –O–C**H**_**3**_), 2.02–1.61 (2H, –C**H**_**2**_– backbone), 1.25–0.51 (3H, –C**H**_**3**_ backbone).

Obtained hydroxyl-functionalized polymer **I** (100 mg) according to previously described method^37–42^ was dissolved in pyridine. Next, the solution was cooled down to 0 °C and α-bromoisobutyrate bromide (25 µL, 0.2 mmol) was instilled. The stirring was continued overnight. Next, the bromoester-functionalized polymer **Ia** was precipitated in cooled water and vacuum dried. ^1^H NMR in Fig. S1 (DMSO-d_6_, δ, ppm): 3.91 (4H, –C**H**_**2**_–O–), 3.68–3.21 (3H, –O–C**H**_**3**_), 1.93 (6H, –(C**H**_**3**_**)**_**2**_Br initiating moiety), 2.02–1.61 (2H, –C**H**_**2**_– backbone), 1.25–0.51 (3H, –C**H**_**3**_ backbone).

#### Synthesis of graft copolymers bearing Cl (example for IV)

The mixture of comonomers ChMA/Cl (1.59 g, 7.6 mmol) and MMA (2.42 ml, 22.9 mmol), methanol (1.5 ml), THF (1.5 ml), bpy (23.8 mg, 0.15 mmol) and initiator **Ia** (48.4 mg, 0.076 mmol of initiating sites) were freeze–pump–thawed twice and CuCl catalyst (7.5 mg, 0.076 mmol) was added. The exposure to air has stopped the reaction after 0.5 hour. The polymer purification was performed by precipitation in chloroform-diethyl ether mixture.

^1^H NMR in Fig. S1 (DMSO-d_6_, δ, ppm): 4.59–4.21 (2H, –C**H**_**2**_–O–), 3.95–3.65 (2H, –C**H**_**2**_–N^+^ and 4H, –C**H**_**2**_–O– from macroinitiator), 3.56 (3H, –O–C**H**_**3**_), 3.44–3.06 (9H, –N^+^–(C**H**_**3**_)_3_), 2.08–1.51 (2H, –C**H**_**2**_–), 1.25–0.51 (3H, –C**H**_**3**_).

#### Synthesis of Sal containing monomer (ChMA/Sal)^33^

The monomer with pharmaceutical anion has been prepared according to previously described procedure^33^. Vacuum dried ChMA/Cl (50 mmol, 10.38 g) was dissolved in 20 ml of water. Then NaSal (52 mmol, 8.33 g) was added. The solution was stirred for 24 hours in room temperature, and then it was extracted three times with chloroform to remove free salicylate salt and monomer with chloride anion. A water phase containing Sal based monomer was concentrated and dried under reduced pressure for 24 hours. Yield 62%.

^1^H NMR (DMSO-d_6_, δ, ppm): 7.66, 7.13, 6.55 (4H, salicylate ring), 6.09 (1H, vinyl), 5.75 (1H, vinyl), 4.54 (2H, –C**H**_**2**_–O–), 3.75 (2H, –C**H**_**2**_–N^+^–), 3.41 (1H, –O**H**), 3.18 (9H, –N^+^–(C**H**_**3**_)_3_), 1.91 (3H, –C**H**_**3**_).

#### Synthesis of graft copolymers bearing Sal (example for VIIa)

The mixture of comonomers ChMA/Sal (0.8 g, 2.59 mmol) and MMA (0.82 ml, 7.76 mmol), methanol (3 ml), THF (2 ml), bpy (32.3 mg, 0.2 mmol) and initiator **Ia** (131 mg, 0.1 mmol mmol of initiating moieties) was freeze–pump–thawed twice and the and CuCl catalyst (10.2 mg, 0.1 mmol) was added. The exposure to air has stopped the reaction after 0.5 hour. The polymer purification was performed by precipitation in chloroform-diethyl ether mixture. ^1^H NMR in Fig. S1 (DMSO-d_6_, δ, ppm): 7.64, 7.13, 6.55 (4H, salicylate ring), 4.57–4.19 (2H, –C**H**_**2**_–O–), 3.95–3.65 (2H, –C**H**_**2**_–N^+^ and 4H, –C**H**_**2**_–O– from macroinitiator), 3.56 (3H, –O–C**H**_**3**_), 3.44–3.06 (9H, –N^+^–(C**H**_**3**_)_3_), 2.08–1.51 (2H, –C**H**_**2**_–), 1.25–0.51 (3H, –C**H**_**3**_).

### Characterization

The molecular weight (M_n_) and dispersity index (Ð) were obtained by size exclusion chromatography (SEC) 1100 Agilent 1260 Infinity with differential refractometer MDS RI Detector. The polymer samples (2 mg/mL) in THF with addition of LiNTf_2_ (1 mmol/L; macroinitiators and Cl based graft polymers) or DMF (Sal contained graft polymers) were separated with sequence of precolumn guard 5 µm (50 × 7.5 mm) and PLGel 5 µm MIXED-C column (300 × 7.5 mm) with a flow rate of 0.8 mL/min at 40 °C. The collected data were processed by Addon Rev. B.01.02 data analysis software (Agilent Technologies) and the linear polystyrene standards (580–300,000 g/mol) were used. Lower values of SEC molecular weights in comparison to those calculated from NMR were a result of higher hydrodynamic volume of polar and non-linear ionic graft polymer samples running slower through the porous columns than hydrophobic and linear polystyrene standards. ^1^H NMR spectra were collected on Varian Inova 300 MHz spectrometer for the samples in DMSO-d_6_ with TMS internal standard at 25 °C. Hydrodynamic diameters d_h_ of polymer particles were measured on Malvern Zetasizer Nano-S90 (4 mW He–Ne ion laser, λ = 633 nm) for samples in deionized water (0.5 mg/mL) at 25 °C ± 0.1 °C. Nanoparticles were visualized by high-resolution transmission electron microscope (TEM, FEI Tecnai G2 Spirit BioTWIN) at 120 kV. The polymer samples in deionized water (0.5 mg/mL) placed on the carbon coated cooper grids (200-mesh) were dried for 2 hours. The measurements by fluorescence spectrophotometry (Hitachi F-7000) were performed for samples of polymer solutions (2.5 × 10^−4^–1.0 mg/mL) containing rhodamine B probe (3.0 × 10^−4^ mol/L, λ_em_ = 550 nm). The excitation spectra of rhodamine B were used to determine the intensity ratio (I_577_/I_575_) which was next plotted against logarithm of the concentration in mg/mL to estimate the critical micellization concentration (CMC) value.

Drug release studies were performed as for linear copolymers reported earlier^43^. Polymers were dissolved in PBS pH = 7.4 (1.0 mg/mL). The sample solution (2 mL) was introduced into a dialysis cellulose membrane bag (MWCO 3.5 kDa), which was placed into a glass vial with 30 mL of PBS and stirred at 38 °C in a water bath. Dialysis was carried out for one week. The buffer solution sample (100 µL) was taken from the released medium at appropriate time intervals to determine the concentration of released drug by UV-Vis spectroscopy at λ = 295–298 nm using standard curve (Fig. S2).

Cell viability assessment was carried out as in previous studies^43^. *In vitro* cytotoxicity was measured for the selected copolymers using a 3-(4,5-dimethylthiazol-2-yl)-2, 5-diphenyltetrazolium bromide (MTT) assay. Bronchial epithelial cells (BEAS-2B) were obtained from American Type Culture Collection (ATCC, Manassas, VA, USA) and Normal Human Dermal Fibroblasts (NHDF) from Lonza (Lonza; Celllab, Warszawa, Poland). All cells were grown in DMEM-F12 medium (Sigma-Aldrich, Germany) supplemented with 10% (v/v) of inactivated fetal bovine serum (FBS) (EURx, Poland) and 1% antibiotics (10000 μg/ml of streptomycin and 10000 units/ml of penicillin) (Sigma-Aldrich, Germany) at 37 °C in humidified atmosphere with 5% CO_2_. Briefly, the human cells were seeded in a 96-well plate at a density of 10 000 cells per well. After 24 h of incubation under standard conditions, a series of polymer solutions (0.007–2.5 mg/mL) was added into wells. After 72 h of incubation, the cytotoxicity was evaluated by measuring of the absorbance at 560 nm with the use of a microplate reader (Epoch, Biotek, Winooski, VT, USA).

Antibacterial activity assessment was performed as in previous studies^43^ against Gramm-negative bacteria species, *Escherichia coli*, from overnight culture in standard liquid LB medium (A&A Biotechnology, Gdynia, Poland). The inoculums of about 2.5 × 10^3^ cells/mL of *E. coli* in respective medium were incubated with selected copolymers in PBS solutions in sterile 96-well microtiter plates, at 37 °C for 24 h, with shaking at 150 rpm. The optical density of the microbial suspensions were measured relative to a control culture, without tested compounds (100% viability) using an microplate spectrophotometer at wavelength λ = 600 nm (Epoch, Biotek, Winooski, VT, USA). Relative viabilities were expressed as % reduction of the viability of microbial cultures compared with controls without tested compounds.

Pro-inflammatory interleukins IL6 and IL8 (RT-qPCR) gene expressions assessment was performed as for analogs of linear copolymers in earlier studies^43^. After completion of the incubation during 72 h with the tested polymer samples at concentration of 2.5 µg/mL each, the culture of BEAS-2B cells was collected by trypsinisation. Supernatant was removed and RNA was isolated from cells with the procedure, by means of phenol-chloroform method of extraction, using the Total RNA Isolation kit (A@A Biotechnology). The efficiency of RNA isolation was assessed by spectrophotometry, and amplification of IL-6 and IL-8 genes (pro-inflammatory genes) was performed with the use of commercially available kits (Real-Time 2xPCR Master Mix SYBR A; A@A Biotechnology) and pairs of primers (Genomed): *i)* IL 6 reverse: AGATCACCTAGTCCACCCCC; IL-6 forward: GTTCTGCCAAACCAGCCTTG; *ii)* IL 8 reverse: ACCAAGGCACAGTGGAACAA; IL-8 forward: GGTGCAGTTTTGCCAAGGAG; *iii)* Reference RPL41 reverse: ACGGTGCAACAAGCTAGCGG; Reference RPL41 forward: TCCTGCGTTGGGATTCCGTG. The quantitative PCR reaction, preceded by reverse transcription (NG dART RT kit, EURx), was performed with the use of thermocycler CFX96 Touch™ Real-Time PCR Detection System (Bio-Rad). The thermal profile of reaction was as follow: 1) 50 °C, 2 min, 2) 95 °C, 4 min, 3) 54 cycles of: 95 °C, 45 sec.; 52,3 °C, 30 sec.; fluorescence reading, 4) 72 °C, 5 min, 5) melting curve from 52 °C to 92 °C (every 0,5 °C at 5 sec), 6) incubation for every sample at 4 °C. The calculation of standardized value of relative gene expression level in an unknown sample was performed in relation to control in accordance with the formula R = 2^−∆∆Ct^ ^44^.

## Results

### Polymer synthesis

Copolymers of HEMA and MMA varied with content of hydroxyl groups (**I**–**III**) were prepared via ATRP and then the hydroxyl groups in HEMA units were esterified to introduce bromoester groups in order to obtain multifunctional macroinitiators (MI, **Ia**–**IIIa**) (Table 1).

**Table 1:** Characterization of macroinitiators (Ia, IIa, IIIa) and their precursors (I–III).

No	[HEMA]_0_ (mol%)	Monomer conversion^a^ (%)	DP^a^	M_n_^b^ (g/mol)	Ð^b^	HEMA or BIEM^a^ (mol%)
**I**	25	57	342	36500	1.52	22
**Ia**	—	—	342	47700	1.61	22
**II**	50	33	198	23000	1.39	53
**IIa**	—	—	198	38600	1.68	53
**III**	75	46	276	33200	1.44	67
**IIIa**	—	—	276	60750	1.87	67

Conditions for **I**–**III**: [HEMA + MMA]_0_:[EBiB]_0_:[CuBr]_0_:[dNbpy]_0_ = 600:1:1:2, monomer:anisol ratio 10:1, temperature 60 °C; **Ia**, **IIa** and **IIIa**: bromoesterification of hydroxyl groups in copolymers **I**, **II** and **III**, respectively (pyridine, rt, stirred overnight); ^a^determined with ^1^H NMR, where DP is degree of polymerization, ^b^determined with SEC (THF, polystyrene calibration), where M_n_ is number average molecular weight of polymer, Ð polymer dispersity.

**Figure 1:**
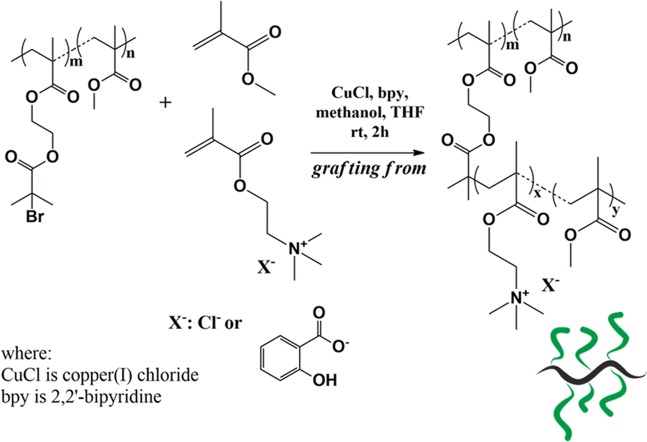
Synthesis of ionic graft copolymers.

In the next step, the resulted in MI with various content of initiating units were applied in the ATRcopolymerization of methacrylates, i.e. ionic ChMA with MMA, to synthesize graft copolymers decorated with Cl or Sal in the side chains (Fig. 1). The Cl contained copolymers with 25 mol% of choline units (**IV**–**VI**) were prepared using macroinitiator with the lowest amount of initiating units (**Ia**) to optimize the reaction conditions (Table 2). Previously applied conditions for synthesis of the linear analogs P(ChMA/Cl-*co*-MMA)^32^ were modified to get the well-defined graft copolymers. The difference was related to the solvent mixture ratio of MeOH/THF, particularly THF quantity (1/1 instead 0.75/0.25), which was higher due to the macroinitiator solubility problem. The polymerization kinetics studies showed that the molar ratio of ionic units to MMA in the side chains remained constant within 3 h.

**Table 2:** Characteristics of Cl contained graft copolymers.

No.	Time (h)	X_M_^a^ (%)	DP_sc_^a^	F_ChMA/Cl_^a^ (mol%)	n_sc_^a^	M_n_ (g/mol)	Ð^b^	d_h_^c^ (nm)	CMC (mg/mL)
**IV**	1	5	20	25	75	235000	1.26	18	0.005
**V**	2	13	50	25		524450	1.32	22	0.005
**VI**	3	19	75	25		706300	1.35	23	0.0005

Conditions: [ChMA/Cl + MMA]_0_:[**Ia**]_0_:[CuCl]_0_:[bpy]_0_ = 100 + 300:1:1:2, methanol:THF 1:1, where methanol:ChMA/Cl = 1 ml:1 g, 40 °C; ^a^determined with NMR, where X_M_ is monomer conversion, DP_sc_ polymerization degree of side chains, F_ChMA/Cl_ is content of ChMA/Cl in side chains, n_sc_ number of side chains, ^b^determined with SEC (THF, polystyrene calibration), where M_n_ is number average molecular weight of polymer, Ð polymer dispersity, ^c^determined with a DLS, where d_h_ is hydrodynamic diameter; CMC is critical micellization concentration.

**Figure 2:**
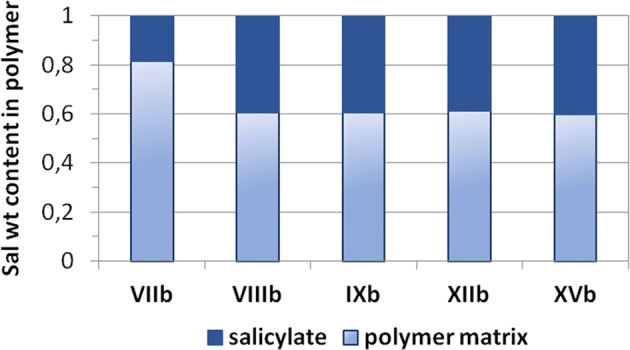
Weight ratio of salicylate to graft polymer matrix.

Direct copolymerization of ChMA/Sal with MMA resulted in the well-defined, water-soluble grafted copolymers with already incorporated biologically active substance via ionic bonding (Table 3). The Sal based graft copolymers (**VII**–**XV**) differed with number and length of side chains (n_sc_ and DP_sc_) as well as composition including the content of ionic units in the side chains (20–91 mol%). The variation of structural parameters let to obtain the well-defined number of ChMA/Sal units. The weight ratio of drug to graft polymer matrix is presented for selected systems in Fig. 2.

**Table 3:** Characterization of Sal contained graft copolymers.

No.	f_ChMA/Sal_ (mol%)	Time (h)	X_M_^a^ (%)	DP_sc_^a^	F_ChMA/Sal_^a^ (mol%)	MI/n_sc_^a^	M_n_ (g/mol)	Ð^b^	d_h_^c^ (nm)	CMC (mg/mL)
**VIIa**	25	0.5	34	17	29.4	**Ia**/75	243500	1.04	60	0.005
**VIIb**		1	48	24	25.0		324000	1.04	28	—
**VIIIa**	50	0.5	12	6	66.7		151400	1.04	73	—
**VIIIb**		1.5	24	12	66.7		256300	1.04	56	—
**IXa**	75	0.5	22	11	90.9		282300	1.05	48	—
**IXb**		3	56	28	71.4		568800	1.1	51	0.01
**Xa**	25	0.5	32	16	25.0	**IIa**/105	289300	1.05	16	—
**Xb**		3	74	37	21.6		603300	1.2	15	—
**XIa**	50	0.5	16	8	50.0		216100	1.05	26	—
**XIb**		1	24	12	50.0		303500	1.06	16	—
**XIIa**	75	0.5	12	6	83.3		205800	1.04	17	—
**XIIb**		3	78	39	74.4		1086000	1.06	22	—
**XIIIa**	25	0.5	22	11	27.3	**IIIa**/185	380900	1.14	24	—
**XIIIb**		3	78	39	20.0		1171200	1.45	56	0.005
**XIVa**	50	0.5	22	11	36.4		419700	1.35	29	—
**XIVb**		3	78	39	33.3		1287600	1.87	33	—
**XVa**	75	0.5	22	11	63.6		554700	1.05	35	—
**XVb**		3	86	43	74.4		2099100	1.28	40	0.005

Conditions: [ChMA/Sal + MMA]_0_:[MI]_0_:[CuCl]_0_:[bpy]_0_ = 50:1:0.5:1, methanol:THF 1,5:1, where methanol:ChMA/Sal = 2.5 ml:1 g, rt; f_ChMA/Sal_ is initial content ChMA/Sal; ^a^determined with NMR, where X is monomer conversion, DP_sc_ polymerization degree of side chains, F_ChMA/Sal_ content of ChMA/Sal in side chains, n_sc_ number of side chains, ^b^determined with SEC (DMF, polystyrene calibration), where M_n_ is number average molecular weight of polymer, Ð polymer dispersity, ^c^determined with DLS, where d_h_ is hydrodynamic diameter; CMC is critical micellization concentration, - means not determined.

### Behavior in aqueous solutions studies

The self-assembling ionic graft copolymers were investigated in aqueous solutions by means of fluorescence spectrophotometry. The stiffness of Cl decorated grafts in macromolecules **IV** and **V** was significantly reduced by increasing length of side chains (**VI**) at the same content of ionic fraction (F_ChMA/Cl_ = 25%), which was evaluated by 10 times lower values of CMC (0.0005 mg/mL) indicating the improved micellization by the long-graft polymer.

**Figure 3:**
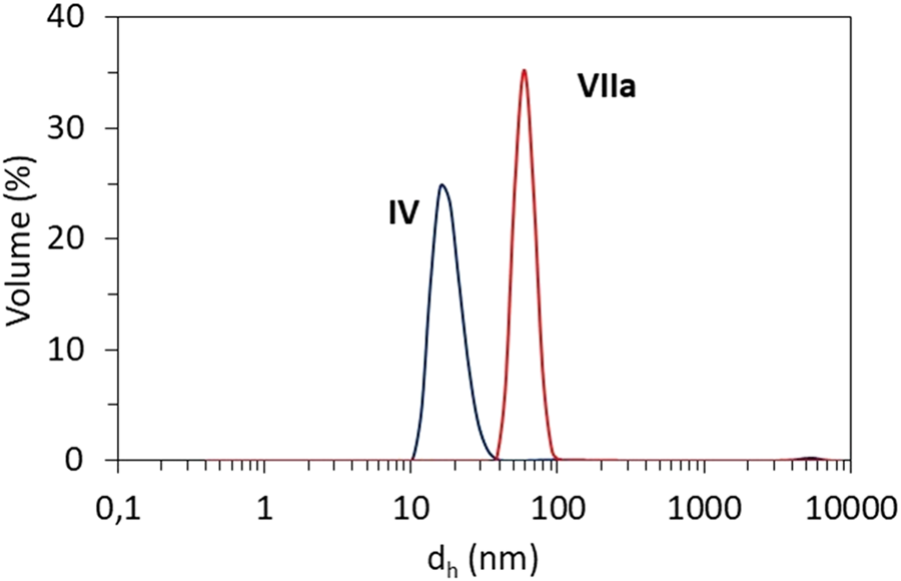
Histograms of particles formed by chloride (**IV**) and salicylate (**VIIa**) containing graft copolymers with comparable DG and anion content for 0.5 mg/mL concentration in deionized water at 25 °C.

Hydrodynamic diameters (d_h_) determined by DLS for the self-assembled copolymers containing chloride anions **IV**–**VI** were similar showing slightly increasing tendency for significantly increasing side chain length (Table 2). Broad range of particle sizes was achieved for Sal based graft copolymers by variety of ionic content, grafting degree (DG), and side chain lengths. Table 3 presents d_h_ values for the main fraction of self-assemblies bearing Sal, which in the most cases reached domination above 82%, although there were some exceptions for **IXb** (60%) and **VIIIa**, **XIIIb** (72%) with more significant tendency to aggregation then the other ones (Table S1). One fraction of nanoparticles was observed only for sample **VIIa** represented by loosely grafted copolymer with low ionic fraction. However, the influence of anion nature (small Cl vs aromatic Sal) was indicated by larger particles for the latter ones, when polymers **IV** and **VII** with similar structural parameters were compared (Fig. 3).

**Figure 4:**
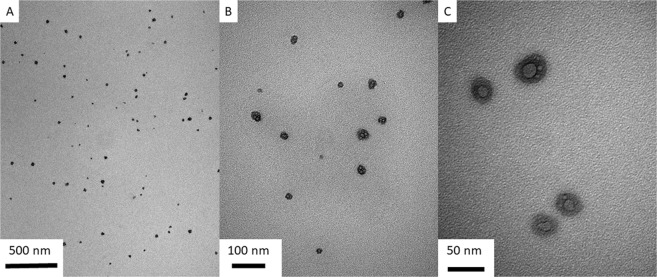
TEM analysis of graft copolymer **XIVb**.

The tendency to formation of superstructures, which in accordance to DLS had sizes below 60 nm, was also confirmed by TEM. The transmission analysis of copolymer **XIVb**, is illustrated by images in Fig. 4, which demonstrate small spheres on the surface of the carbon film. Their sizes reaching ~30 nm and no larger aggregates proved relatively low affinity to superaggregation. Interestingly, on the greater magnification the core-shell superstructures can be easily distinguished (Fig. 4C).

### *In vitro* release studies

**Figure 5:**
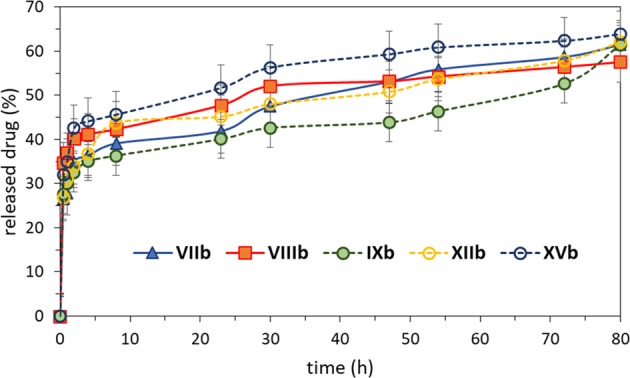
Salicylate release profiles for selected graft copolymers in PBS pH 7.4 at 38 °C, number of repetitions is 3.

The synthesized graft polymers were subjected to release studies, which were performed using dialysis method in PBS imitating conditions of human blood (that is pH 7.4 and temperature 38 °C). Figure 5 shows the release profiles of anionic drug for the selected graft copolymers. Burst release of drug up to 35 to 44% was exhibited for all samples at initial 4 h, and then the release slowed down. Next portion of Sal (20–30%) was removed out of the polymer matrix within 70 hours.

Additionally, various kinetic models were used to describe the drug release in order to define the main mechanism of this process. The release correlation coefficients were determined showing the best fit with Higuchi model (R^2^ = 0.91–0.97) in contrary to zero order and first order models (Supplementary Materials Fig. S3 and Table S2).

### Biological studies

**Figure 6:**
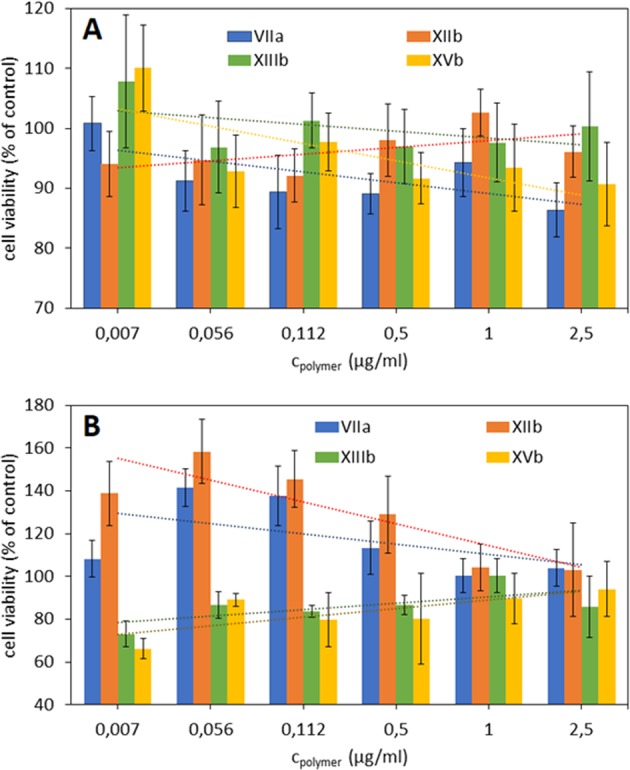
Relative viabilities: 10 × 10^3^ of BEAS-2B (**A**) and NHDF (**B**) cells seeded in respective medium incubated with PBS solutions of the selected graft copolymers and control NaSal in 96-well microtiter plates in a humidified atmosphere containing 5% CO_2_ at 37 °C for 72 h.

In the present studies cytotoxic activity of the synthesized ionic graft copolymers was investigated towards human bronchial epithelial cells (BEAS-2B) and normal human dermal fibroblasts (NHDF). The proliferation of cells evaluated by MTT assay, was performed at various solution concentrations of graft copolymers containing Sal anions in choline units exhibiting very low toxic effect or no cytotoxicity (Fig. 6). Reported in the literature IC_50_ of pure salicylic acid (SalA) solutions tested against human normal and cancer cell lines depended on the time of incubation and used doses. Routinely applied for cytotoxicity MTT or MTS assays delivered different results for tissue specific biological responses. Pure SalA used as control in *in vitro* MTS assay within 24 hours against normal fibroblasts 3215 LS was estimated at value IC_50_ = 1.14 mM^45^, whereas for epithelial cancer cell line A549 a IC_50_ = 6 mM was reported^46^. In our studies on NHDF cells, the highest used concentration of Sal did not reach the lowest reported IC_50_ = 1.14 mM (tested doses of 10 µg/ml of copolymers containing Sal was about 15 times lower than IC_50_ = 1.14 mM on fibroblasts 3215 LS). This comparison indicates that the tested copolymers at highest doses were nontoxic against normal human cell lines, NHDF and BEAS-2B in a significant manner.

**Figure 7:**
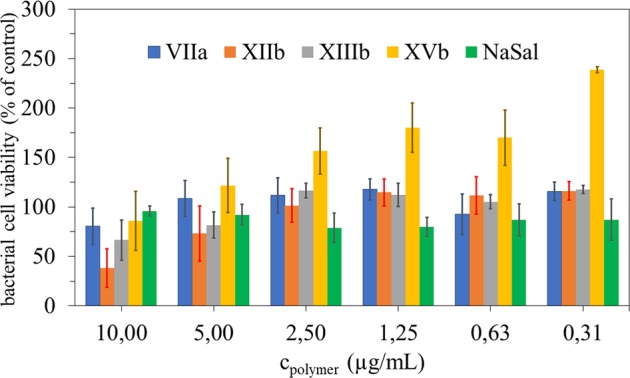
Relative viabilities: inoculum about 2.5 × 10^3^ cells/ml of *E. coli* in respective medium incubated with PBS solutions of the selected graft copolymers in 96-well microtiter plates at 37 °C for 24 h.

As a biological model the wild type of *Escherichia coli* was selected due to its commonness and the fact of its presence in human body. The synthesized graft copolymers with ChMA/Sal units in the side chains exhibited very weak antibacterial activity, which was observed only for higher polymer concentrations (Fig. 7). Effects of low concentrations of SalA could induce the development of tolerance to antimicrobial agents, such as antibiotics, however the mode of action is still not sufficiently documented^47^. Similar concentrations of SalA to Sal in our copolymers (1 µg/mL) were efficient in bacterial viability reduction for many bacterial streams tested for 24–72 hours^48^. Some of the SalA derivatives could be not efficient in their antimicrobial activities, because of insufficient doses and chemical structure modifications, in such situations the stimulatory effects could be present instead of toxicity^49^.

**Figure 8:**
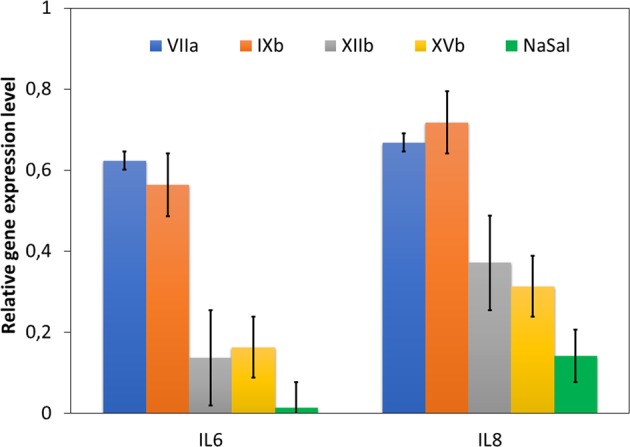
Relative gene expression level, for pro-inflammatory IL 6 and IL 8 cytokines in BEAS-2B cells after 72 h of incubation [R = 2^−∆∆Ct^]. NaSal at dose of 2.5 µg/ml was used as additional control.

Anti-inflammatory effect of the graft copolymers was evaluated on the transcriptional level using gene expression measurements for interleukins IL 6 (pro-inflammatory cytokine) and IL 8 (chemokine-macrophages chemoatractants). These interleukins are mainly expressed by epithelial cells and they are known as inflammation state markers^50^. The results are presented as relative to the reference gene expression of RPL41 in Fig. 8. According to general rules the ratio increased above 1 means the activation of pro-inflammatory pathways, which emerged after 72 h of incubation with cytokines production.

## Discussion

ATRP as the controlled synthesis method and “grafting from” multifunctional MIs (Table 1) provided the well-defined graft copolymers, which were decorated with various anions, including the biologically active ones like Sal. The polymeric systems with adjustable number of side chains, their length and composition seem to be convenient to regulate the drug amount to a sufficient level by proper conditions of polymerization, especially in the design of polymerized ionic drug carriers. The graft copolymers with chloride anions, which content was corresponding to the initial content of ionic monomer in reaction mixture (Table 2), can be used as matrices for exchange of chloride into the pharmaceutical one to achieve biological activity. However, in the strategy of post-polymerization modification the drug-polymer ratio is strongly dependent on the efficiency of exchange reaction. This disadvantage can be omitted by preparation of biologically active ionic monomer, like ChMA/Sal. Generally, after half an hour of polymerization the content of Sal based units in grafts was higher than the initial content of ionic monomer in the reaction mixture, but when the reaction was continued up to 1–3 h this relation was changed to similar or slightly lower ionic content in side chains, for example 29% to 25% for **VII** or 50% to 50% for **XI** (Table 3). However, there was one exception for copolymer **XV**, where the ratio of ionic to neutral fraction was increased with the polymerization time (64% to 74%) indicating lower relative reactivity of monomer with large Sal counteranion. In this case the steric hindrance generated by the highest DG was combined additionally with the highest content of rigid charged units.

In contrast to the synthesis of analogous linear copolymers^33^, the polymerization conducted at 40 °C was very fast and poorly controlled leading to polymers with very high molecular weights (up to 5 × 10^6^ g/mol) and high dispersity indices (above 2.5). Fortunately, this problem was fixed applying room temperature (rt) in the polymerization, which yielded copolymers with molecular weights varied from 1.0 × 10^5^ g/mol to 1.5 × 10^6^ g/mol and significantly narrow its distributions < 1.1 for most of the proposed systems. Higher dispersities of densely grafted copolymers **XIII**–**XV**, especially that with longer side chains (**Ð = **1.3–1.9 at DP_sc_~40 and DG = 74%), were probably caused by steric hindrance, which can be responsible for increased ability of the crowded Sal to coordinate the catalyst.

Because of amphiphilic nature the graft copolymers P((BIEM-*graft*-P(ChMA-*co*-MMA))-*co*-MMA) were self-assembled in aqueous solution. Both types of graft copolymers with similar content of ionic units (Cl^−^ vs Sal^−^, *i.e*. **IV** vs **VIIa**) in the side chains and grafting degree (22%) or with different grafting degree (22% vs 67%, **V** vs **XIIIb**) showed no influence of counterion nature on CMC (0.005 mg/mL). However, the increased content of ionic units (F_ChMA/Sal_ = 29% vs 71%) at the lowest grafting degree and similar graft length in samples **VIIa** vs **IXb** provided tendency to micellization at twice higher concentration for the latter one probably due to stronger rigidity and repulsion effect. These results are in contrary to **XIIIb** vs **XVb**, what suggests that macromolecules characterizing with higher grafting degree and relatively short side chains (not exceeding 50 repeating units) can be used at smaller amount to form self-assemblies at critical value.

The nanoparticles of the self-assembling graft copolymers, which were detected by DLS, reached smaller sizes in comparison with that formed by previously investigated linear P(ChMA-*co*-MMA)^33^ (<100 nm vs 150–300 nm). In the case of graft copolymers the hydrophobic backbone was shrunk yielding core, which was covered by layer of more or less entangled side chains depending on the ionic content. The extension of side chains caused formation of smaller or comparable sizes of particles for that with shorter grafts in the case of copolymers with DG 20% and 53% (**VII**–**XI**), whereas this correlation is opposite at larger content of pharmaceutic anion, that is at DG 53% with 75% of Sal content (**XII**) or DG 74% (**XIII**–**XV**). Results obtained from DLS were confirmed by TEM. Spherical nanoparticles with low diameters were visualized without any staining (Fig. 4). Lack of aggregation might suggest that CMC results are connected with intramolecular assembly more than with assembly of more than one macromolecule.

As it was proposed in our previous studies on the linear copolymers of ChMA/Sal^33^, the delivery of Sal is possible in PBS media containing phosphate anions, which are capable to replace Sal anions in polymer matrix and the release is driven by the concentration gradient and diffusion. The graft topology of carriers may be advantageous for delivery of larger amount of pharmaceutical anions. Drug-polymer weight ratio (Fig. 2) shows the lowest value for **VIIb** and then it was increased to 40% as the highest achievable ratio in the other studied Sal based graft copolymers independently on grafting density, ionic content and length of side chains. Surprisingly, Sal content in the graft copolymers (0.2–0.4) was similar to that provided by the linear ionic polymer matrices (0.3–0.4)^33^.

The release experiments did not show any significant differences (Fig. 5). The series of copolymers with the same DG, but various hydrophilic-hydrophobic ratios demonstrated very similar profiles for **VIIb**, **VIIIb** and slightly slower release by **IXb** yielding for example 51–53% and 40% of released Sal after 47 h. Another series of polymers containing comparable content of ionic units, but varied with DG, **IXb**, **XIIb**, and **XVb**, also resulted in small differences in the release rates ranging in approximately 10%, which were proportionally increased with density of side chains (40%, 51% and 60%, respectively within 47 h). These results indicated that in fact the release effect did not depend on ionic graft composition, but it could be slightly adjusted (with approximately 20%) by grafting density. The fastest and largest amount of released Sal can be achieved by designing copolymer with both dominated ionic content and very high grafting degree, which evidently generated both steric and repulsion effects between side chains closely distributed among the backbone. Additionally, the best fitting of release profiles to Higuchi model confirmed the diffusion controlled mechanism. The release profiles are similar to those exhibited in our earlier investigations for linear copolymers of ChMA/Sal^33,43^, with slightly lower “burst” effect and 10% higher final release in the case of comb-like copolymers. However, comparing both types of Sal contained systems the graft polymers with hydrophobic backbone seem to be more advantageous due to smaller sizes of nanoparticles, which should be helpful to provide effective biodistribution of ionic drug and improved pharmacokinetic properties.

Copolymers bearing cationic moieties are often used for biological and medical purposes, especially those that have low cytotoxicity are increasingly sought^51^. The introduction of choline as a cationic group into the polymer matrix caused lowering of negative effects of the reported systems^52^. The cell viabilities slightly decreasing with the increase of polymer concentration was ranged from 86% to 110% of the control for BEAS-2B (Fig. 6A), and from 65% to 160% of the control for NHDF (Fig. 6B). However, this tendency was opposite for NHDF cells treated with high DG copolymers (**XIIIb** and **XVb**), which let to conclude the influence of grafting degree on behavior of this type cell line in contrary to BEAS-2B cells. Low DG seems to be beneficial for NHDF cells resulting in slight stimulation and increased cell growth for concentrations below 1 µg/ml. However, at lowest doses we have found a stimulatory potential for some of tested copolymers, more effective in NHDF than in BEAS-2B cell line. The cytotoxicity was assayed followed by long-term MTT assay, within 72 hours of incubation with copolymers containing different doses of Sal and obtained cellular effects were not linear. Such activities of tested copolymers, observed during long-term assays are known from the literature, where 1,3 and 5 mM doses of acetyl-SalA were effective and reduced viability of human lymphocytes within 48 hours of assays, however lower doses were more effective than higher^53^. Such non-linear to dose-depended activities are known as hormetic effects – lower doses could be also stimulatory and improve cellular proliferation, whereas higher are neutral or toxic^54,55^.

SalA is well known as slightly toxic towards bacteria^56^, thus polymers bearing these anions should also exhibit antibacterial properties. This potential activity was studied on the wild type *E. coli* bacteria. Lower concentrations of polymers provided rather stimulating effects on bacteria than antibacterial properties (Fig. 7). Interestingly the grafting density and side chain length influence on the bacterial cell viability was detected. Copolymer **VIIa** with low DG and ionic fraction, including low Sal and cationic moieties content, was able to kill up to 20% of cells at the highest concentration. The grafting density enlarged to 50% in copolymer **XIIb** corresponded to antibacterial activity already at 5 µg/ml concentration killing 30% of bacteria, which was improved to 62% at higher concentration. However, the highly grafted copolymer **XIIIb** was less effective than **XIIb**, resulting 19% of killed cells at 5 µg/ml concentration and 33% at 10 µg/ml. At concentrations below 2.5 µg/ml stimulating effect allowed for bacterial growth between 101 to 118% for copolymers **VIIa**, **XIIb** and **XIIIb**. In the case of copolymer **XVb**, which had 75% of grafting density and 75% of Sal content, only 14% of cells was killed at the highest concentration, whereas for other concentrations only stimulating effect was monitored yielding bacterial growth from 121% to 240% at the lowest concentration. No antibacterial activity might be explained by hiding of Sal and cationic moieties due to high density and rigidity of grafting. Antibacterial activity of SalA is known since ancients, concentration of 5 mM used for 1 hour against proliferation of Gramm negative *Escherichia coli* streams seemed to be effective and stopped bacterial proliferation and induced other cellular effects (changes in operons and genes expression levels)^47,57^. For some of our tested copolymers a stimulatory action was observed in *E. coli* during 24 hours of viability assays. Small concentrations of Sal, lower than for SalA reported in literature, could induce bacterial proliferation or induce chemo-resistance, without inhibitory effects^47^.

Anti-inflammatory properties of the obtained systems were also studied on interleukins IL 6 (pro-inflammatory cytokine) and IL 8 (chemokine-macrophages chemoatractants). Gene expression of two interleukins in BEAS-2B cells demonstrated dependency on the content of Sal bind to polymer matrix. Each copolymer was able to stop the inflammation state in the cells and to decrease mRNA level (Fig. 8). In comparison to samples **VIIa** and **IXb**, the copolymers **XIIb** and **XVb** exhibited higher activity, reducing cytokines production on the transcriptional level for IL8 up to 37% and 31%, respectively. In the case of IL6 this effect was even more spectacular reducing level up to 15%. The SalA is also known as anti-inflammatory agent, because of action *via* oxidative stress reduction and pro-apoptotic pathway inhibition^58,59^. Evolutionary conserve apoptotic pathway can be regulated by NFĸB feedback loop, and SalA at doses 1–20 mM influencing on cellular death^58^. Release of interleukin-1β (IL-1β), and Il-6, as a pro-inflamatory stimulating cytokine, could be reduced significantly by SalA^60^, also in neutrophils in *in vivo* asthma mouse model^61^. In our studies we have evaluated inflammatory cytokines level (IL-6 and IL-8) on the transcriptional level^62^, when the first step of physiological cellular response to the tested copolymers. We haven’t observed an activation of inflammation process, what is a good prediction for potential application of copolymers, which are neutral against normal human cells. Literature reported a dose-dependent impact of SalA on IL-6 and IL-8 cellular release from blood cells, whereas high concentrations above 10 mM reduced IL-6 and induced IL-8 production^63^. We didn’t reach such high concentrations during formulation procedure, and the discussed copolymers did not induce an inflammation process in normal fibroblasts NHDF, and epithelial cells BEAS-2B.

## Conclusions

The design of new carriers for delivery of biologically active anions was based on three series of grafted copolymers, which were varied by grafting degree, side chain length and the content of ionic units. However, densely grafted copolymers have demonstrated higher dispersity induces due to steric hindrance of rigid side chains with charge repulsion. Formation of core-shell nanoparticles (15–60 nm) with a slight tendency to aggregation was detected by DLS and visualized by TEM. Release studies indicated the “burst” effect in initial 4 hours, and then the process was slowed down yielding the final Sal release in the level of 60% within 3 days. A biological potential, evaluated against eukaryotic human normal cells (NHDF and BEAS-2B) showed low or even no toxicity, what confirms possible application of tested compounds as novel-carriers. Viability and anti-inflammatory assays resulted with tissue bio-compatibility, observed as low viability reduction or no inflammatory stated induction in cells after treatments. BEAS-2B did not produce pro-inflammatory cytokines (IL-6 and IL-8), at the transcriptional level. Activity against prokaryotic organism showed dose-dependent toxicity of tested compounds, what is a good prognostic for antibacterial mode of action.

## Supplementary information

Supplementary Info
