# Supplementary material for: Choline supported poly(ionic liquid) graft copolymers as novel delivery systems of anionic pharmaceuticals for anti-inflammatory and anti-coagulant therapy
Source: Sci Rep. 2019 Oct 8;9:14410. doi: 10.1038/s41598-019-50896-5 (PMC6783615; doi:10.1038/s41598-019-50896-5)
Supplement: Supplementary file 1 — Supplementary Info [file 41598_2019_50896_MOESM1_ESM.docx]

**SUPPLEMENTARY INFORMATION**

**Choline supported poly(ionic liquid) graft copolymers
as novel delivery systems of anionic pharmaceuticals
for anti-inflammatory and anti-coagulant therapy**

***Rafał Bielas^1^, Anna Mielańczyk^1^, Magdalena Skonieczna^2,3^, Łukasz Mielańczyk,^4^
and Dorota Neugebauer^1*^***

^1^Department of Physical Chemistry and Technology of Polymers, Faculty of Chemistry, Silesian University of Technology, Strzody 9, 44-100 Gliwice, POLAND

^2^Biosystems Group, Institute of Automatic Control, Faculty of Automatics, Electronics, and Informatics, Silesian University of Technology, Akademicka 16, Gliwice, Poland

^3^Biotechnology Centre, Silesian University of Technology, Krzywoustego 8, Gliwice, Poland

^4^Department of Histology and Cell Pathology, School of Medicine with the Division of Dentistry in Zabrze, Medical University of Silesia, Jordana 19, 41-808 Zabrze, Poland

*email: dorota.neugebauer@polsl.pl

**Content:**

1. Conformation of polymer structures by ^1^H NMR in DMSO-d_6_ (Figure S1)
2. Standard curve for determining the release of salicylate by UV-vis (Figure S2)
3. E. coli viability 24h after NaSal addition (Figure S3)
4. DLS studies of nanoparticles (Table S1)
5. Kinetic models of release: (a) I order and (b) Higuchi model (Figure S3)
6. Correlation coefficients for kinetic models (Table S2)


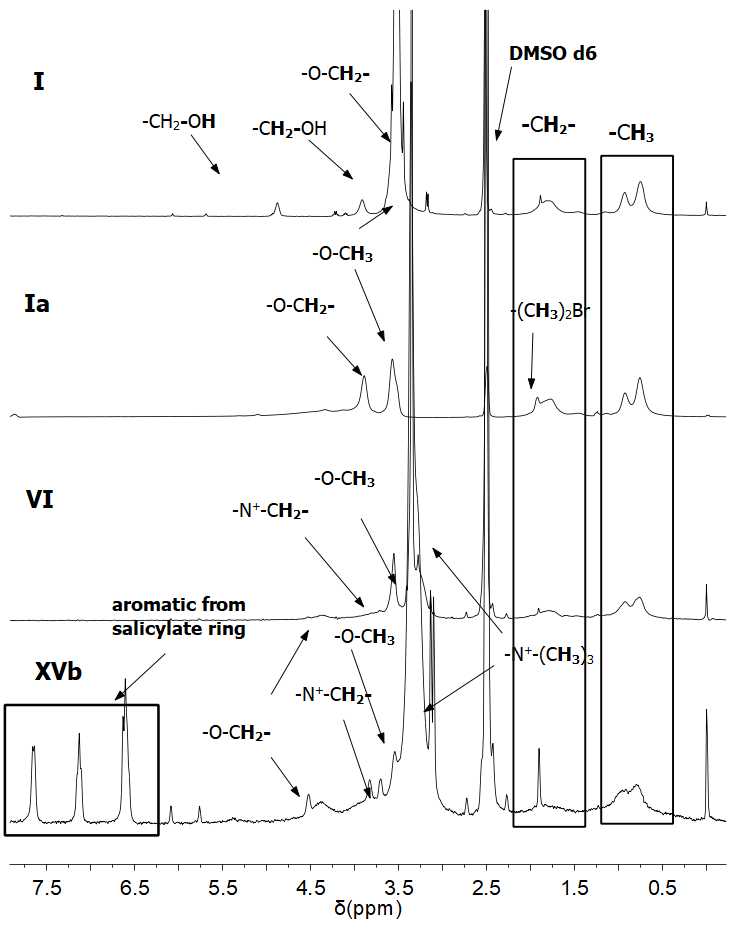


**Figure S1.** ^1^H-NMR spectra of hydroxy-functionalized copolymer (macroinitiator precursor **I**), bromoester-functionalized copolymer (macroinitiator **Ia**), graft copolymer with Cl^-^ (**VI**), and graft copolymer with Sal^-^ (**XVb**) in DMSO-d_6_


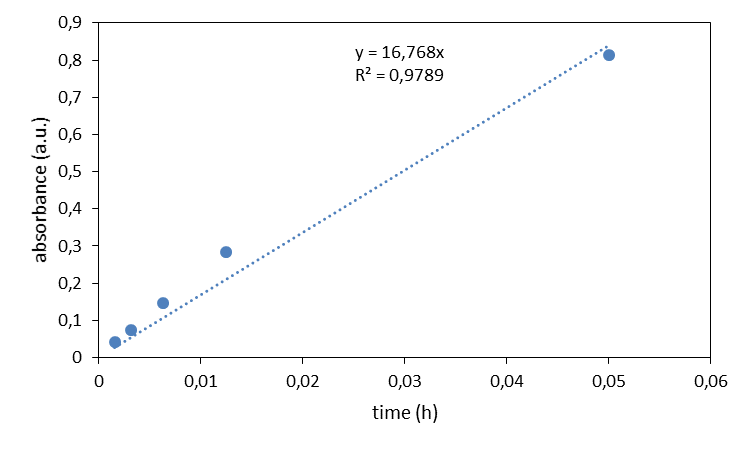


**Figure S2.** Standard curve for determining the release of salicylate by UV-vis

**Figure S3.** Relative viabilities: inoculum about 2.5 x 10^3^ cells/ml of *E. coli* in respective medium incubated with PBS solutions of the control NaSal in 96-well microtiter plates at 37 ^o^C for 24 h.

**Table S1.** Sizes of particles based on Sal containing graft copolymers by DLS

| **Sample No.** | **Hydrodynamic diameter (nm)**  **percent amount of fraction in bracket** | | |
| --- | --- | --- | --- |
|  | **Signal #1** | **Signal #2** | **Signal #3** |
| **VIIa** | 60 (100%) | - | - |
| **VIIb** | 28 (98.4%) | 229 ( 1.6%) | - |
| **VIIIa** | 73 (73.3%) | 570 (26.7%) | - |
| **VIIIb** | 56 (82.3%) | 5178 (17.7%) | - |
| **IXa** | 26 (89.2%) | 112 (10.8%) | - |
| **IXb** | 51 (59.9%) | 319 (40.1%) | - |
| **Xa** | 16 (94.2%) | 79 ( 5.0%) | 360 (0.8%) |
| **Xb** | 15 (96.7%) | 65 ( 3.1%) | 245 (0.3%) |
| **XIa** | 26 (89.2%) | 112 (10.8%) | - |
| **XIb** | 16 (97.3%) | 97 ( 2.7%) | - |
| **XIIa** | 17 (99.0%) | 5207 ( 1.0%) | - |
| **XIIb** | 22 (99.1%) | 100 ( 0.9%) | - |
| **XIIIa** | 24 (95.6%) | 105 ( 4.1%) | 353 (0.3%) |
| **XIIIb** | 56 (72.3%) | 315 (27.7%) | - |
| **XIVa** | 29 (90.7%) | 172 ( 4.0%) | 772 (5.3%) |
| **XIVb** | 33 (86.2%) | 323 (13.8%) | - |
| **XVa** | 35 (87.8%) | 172 ( 4.7%) | 828 (7.5%) |
| **XVb** | 40 (94.6%) | 222 ( 5.4%) | - |


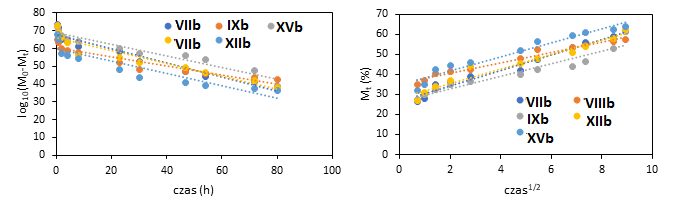


**Figure S3.** Kinetic models of Sal release: (a) I order and (b) Higuchi model

**Table S2.** Correlation coefficients for kinetic models

|  | **R^2^** | | |
| --- | --- | --- | --- |
| **Sample** | **0 order** | **I order** | **Higuchi model** |
| **VIIb** | 0,6943 | 0,9245 | 0,9730 |
| **VIIIb** | 0,4879 | 0,8887 | 0,9745 |
| **IXb** | 0,6508 | 0,9324 | 0,9135 |
| **XIIb** | 0,6435 | 0,8933 | 0,9567 |
| **XVb** | 0,5628 | 0,8410 | 0,9459 |
